# 4423 Impact of Gender on High On-Treatment Platelet Reactivity (HPR) and Major Adverse Cardiovascular Events (MACEs) in Caribbean Hispanic patients using Clopidogrel

**DOI:** 10.1017/cts.2020.336

**Published:** 2020-07-29

**Authors:** Hector Jose Nunez Medina, Jorge Duconge, Luis A. Velez, Laura I. Fernandez, Orlando Arce

**Affiliations:** 1 University of Puerto Rico; 2 University of Puerto Rico Medical Sciences Campus; 3 Universidad Central del Caribe

## Abstract

OBJECTIVES/GOALS: The use of P2Y12 receptor inhibitors like Clopidogrel is crucial in the prevention of thrombotic events in patients with coronary artery disease, peripheral arterial disease, and cerebrovascular disease. Variation in the level of platelet inhibition is present in many patients, and it is associated with the occurrence of major adverse cardiovascular events (MACEs). The term High-on treatment platelet reactivity (HTRP) is used to describe impaired antiplatelet inhibition while on Clopidogrel. Multiple factors have been associated with the presence of HTPR in patients with CAD and PAD, including CYP2C19 loss of function polymorphism, drug-drug interactions, and medical comorbidities. Gender differences are another factor that might influence the levels of platelet inhibition while on Clopidogrel and hence, HTPR. Differences by Gender exist in platelet biology, count, and activation. The evidence for the influence of Gender in HTPR is limited, but a possible association has been described. In this study, we described the association of Gender with HTPR and Major Adverse Cardiovascular Events (MACEs) occurrence. The data is from a sample of Hispano-Caribbean patients on Clopidogrel therapy alone or in combination with Aspirin that were retrospectively evaluated from an ongoing trial in Puerto Rico. The result of this study provided evidence of the influence that Gender has on antiplatelet therapy function and MACEs occurrence. METHODS/STUDY POPULATION: The population in the study consisted of Hispano-Caribbean patients using Clopidogrel alone or in combination with Aspirin for coronary artery disease, peripheral arterial disease, or cerebrovascular disease. The sample was obtained from multiple hospital institutions with cardiovascular services in Puerto Rico during the years 2016-2019. Patients were part of the ongoing trial, “Adopting a precision medicine paradigm in Puerto Rico: leveraging ancestral diversity to identify predictors of Clopidogrel response in Caribbean Hispanics.” The sample size consisted of 150 patients. Participants were recruited during routine medical care, pre-admission evaluation for elective cardiac procedures, or during hospitalization in the participating institutions. Platelet reactivity testing was performed with the system Verify Now® to determine PRU values, and High on-treatment platelet reactivity was defined as PRU ≥208. One year after recruitment, the patients were re-evaluated for the occurrence of MACEs. The association of the variables HTPR, occurrence of MACEs, and Gender were assessed using logistic regression in addition to the role of HTPR and Gender for predicting MACE occurrence. The analysis was done using the statistic software Intellectus ©. RESULTS/ANTICIPATED RESULTS: The sample consisted of 67 females and 83 males with and Mean age of 67.87 years and 61.11 years, respectively. The prevalence of HTPR in the sample was 32.67 % (n = 49) with 36% (n = 24) for females, and 30%(n = 25) for males. The mean PRU values were 179.54 for females and 170.81 for males. The percentage of MACEs one year after recruitment was 29.33 % (n = 44) with 43% on females (n = 19), and 57% on males (n = 25). Logistic regression for Gender predicting HTPR was non-significant with a χ2(2) = 0.55, p = .758, and McFadden R^2^ = 0.00. Also, logistic regression for the effects of Gender and HTPR on the Odds of MACEs occurrence was not significant based on a model with an alpha of 0.05, χ2(2) = 1.99, p = .370, and McFadden R^2^ = 0.01. DISCUSSION/SIGNIFICANCE OF IMPACT: The sample consisted of 67 females and 83 males with and Mean age of 67.87 years and 61.11 years, respectively. The prevalence of HTPR in the sample was 32.67 % (n = 49) with 36% (n = 24) for females, and 30%(n = 25) for males. The mean PRU values were 179.54 (±70.42) for females and 170.81(±64.89) for males. The percentage of MACEs one year after recruitment was 29.33 % (n = 44) with 43% on females (n = 19), and 57% on males (n = 25). Logistic regression for Gender predicting HTPR was non-significant with a χ2(2) = 0.55, p = .758, and McFadden R^2^ = 0.00. Also, logistic regression for the effects of Gender and HTPR on the odds of MACEs occurrence was not significant based on a model with an alpha of 0.05, χ2(2) = 1.99, p = .370, and McFadden R^2^ = 0.01.

